# Positive effects of the tea catechin (-)-epigallocatechin-3-gallate on gut bacteria and fitness of *Ectropis obliqua* Prout (Lepidoptera: Geometridae)

**DOI:** 10.1038/s41598-019-41637-9

**Published:** 2019-03-22

**Authors:** Yong Zhang, Tianyu Zhao, Jundan Deng, Xiaomin Zhou, Zhenzhen Wu, Qingqing Su, Longwa Zhang, Yanhua Long, Yunqiu Yang

**Affiliations:** 1 State Key Laboratory of Tea Plant Biology and Utilization, Anhui Agricultural University, Hefei, 230036 China; 2 School of Forestry and Landscape Architecture, Anhui Agricultural University, Hefei, 230036 China; 3 School of Life Sciences, Anhui Agricultural University, Hefei, 230036 China

## Abstract

*Ectropis obliqua* Prout (Lepidoptera: Geometridae) is the most devastating insect pest of tea plants in China and infests thousands of hectares of tea plantations in China annually. (-)-Epigallocatechin-3-gallate (EGCG) is a major phenolic compound in tea leaves and has a strong antibacterial function. Here, we show that EGCG can effectively improve the fitness of *E*. *obliqua* larvae and present the reason by which EGCG promotes larval fitness. In this study, we compared the fitness difference among Control, Antibiotic and Treatment of larvae. The fitness of larvae treated with EGCG and antibiotic was similar and better than that of control group. We also demonstrated that EGCG treatment could significantly reduce species richness and abundance of gut bacteria in *E*. *obliqua* larvae. Hence that we speculate that EGCG promotes larval fitness and is associated with ECGG antimicrobial activity. In short, our study provides evidence of the *E*. *obliqua* larvae have adapted to secondary compounds found in tea leaves, and may even benefit from these compounds. Our study also contributes to a greater understanding of the reason involved in plant–insect interactions.

## Introduction

Interactions between host plants and herbivorous insects are often mediated by plant secondary compounds, especially alkaloids, terpenoids, and phenolics, which are secondary metabolites that are common among plants^1^. Plant secondary compounds can be toxic to insects or benefit the fitness of insects. For example, the pine weevil feeds on conifer tissues rich in terpenoid resins (primarily monoterpene olefins and diterpene resin acids) that are toxic to many insect herbivores. Diterpene treatment can facilitate pine weevils to lay more eggs and improve the hatching rate of those eggs. And this study also found that the gut symbionts of pine weevil contribute towards host fitness, but not by detoxification of diterpenes, as these compounds do not show toxic effects with or without antibiotics^2^.

Decades of research have shown that insects do not interact with plants in isolation, but together with their gut microbes^3,4^. These gut symbionts can help insects degrading complex dietary polymers or supplementing essential nutrients^5^. Recently, extensive studies illustrated that some gut symbionts can facilitate herbivory insect fitness by detoxifying, or being resistant to, plant toxin^6,7^. For example, gut bacteria *Rothschildia lebeau* of saturniid moths *Automeris zugana* provide gelatinase, caseinase and chitinase activity. And these bacterial enzymatic activities might become especially important for efficient food digestion by the host insect during periods of food shortage^8–10^. A metagenome study reveled that phenol-degrading gene in the gut bacterial (i.,e *Enterobacter asburiae* and *Enterobacter cloacae*) of larvae of the diamond back moth *Plutella xylostella* (Lepidoptera: Plutellidae), which can help host degradation of the harmful phenol^11^.

Tea plant (*Camellia sinensis* (L.) O. Kuntze), a long-lived (i.e., perennial) evergreen woody plant, is an important economic crop in China^12^. Tea leaves contain high volumes of both constitutive and inducible chemical compounds, such as (-)-Epigallocatechin-3-gallate (EGCG), that affect food choice of insect species and influence insect diet breadth. EGCG is a catechin monomer found in tea leaves and accounts for 30% to 60% of the total phenolic compounds in tea leaves^13,14^. EGCG has strong antibacterial function in some animals and demonstrated antibacterial activity^15^.

Tea leaves are a primary host for a suite of insect herbivores, including *Ectropis obliqua* Prout (Lepidoptera: Geometridae). *E*. *obliqua* has been the main focal organism for scientific research due to its economic and ecological importance^16^. *E*. *obliqua* larvae consume large quantities of tea leaves in a short period of time and infest thousands of hectares of tea plantations in China annually^17^. However, *E*. *obliqua* larvae are susceptible to infection by pathogens, parasites and viruses, a trait that can be exploited for biological control. For example, in agriculture, Beauveria bassiana and *E*. *oblique* nucleopoly hedrovirus (EoNPV) are commonly used to control *E*. *oblique*^18^. *E*. *obliqua* larvae necessarily ingest large quantities of EGCG during consumption of tea leaves so, here, we investigated the effect of EGCG on fitness of *E*. *obliqua* and its reason of action.

## Results

### Effects of EGCG on the fitness of E. obliqua

**Figure 1:**
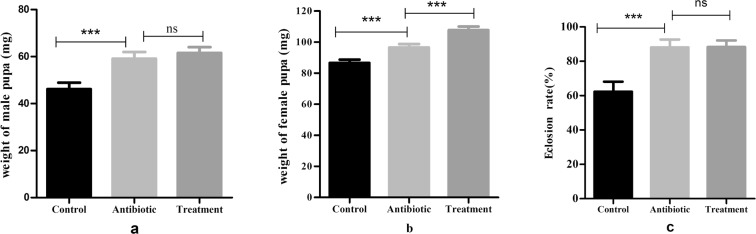
Effects of EGCG on the fitness of *E*. *obliqua* (mean ± SD, n = 7). An unpaired two-tailed *t*-test was performed to assess the significance of differences between groups unless otherwise stated. *P < 0.05; **P < 0.001; ***P < 0.0001. Control: larvae fed on artificial diet; Antibiotic: larvae fed on artificial diet with 300 µg/ml antibiotic solution suppleent; Treatment: larvae fed on artificial diet with 1% EGCG suppleent. a: weight of male pupa; b: weight of female pupa; c: Eclosion rate.

**Figure 2:**
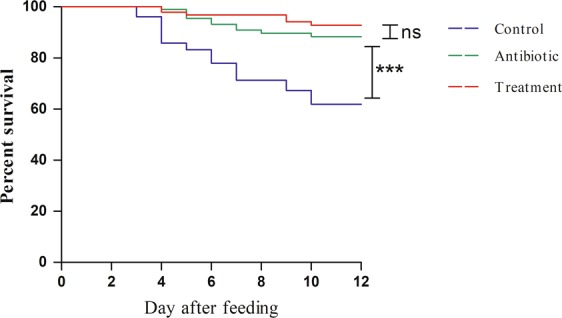
Comparison of survival curves of three larvae groups. The log-rank (Mantel-Cox) test was used to evaluate the significance of differences between groups, ***P < 0.001. Control: larvae fed on artificial diet; Antibiotic: larvae fed on artificial diet with 300 µg/ml antibiotic solution suppleent; Treatment: larvae fed on artificial diet with 1% EGCG supplement.

There were significant differences in the fitness of *E*. *obliqua* between the Control and Treatment. *E*. *obliqua* eclosion rates were significantly higher in the Treatment compared with the Control (Control (mean ± s.d.): 62.86 ± 7.01% vs. Treatment: 88.28 ± 2.18%; Student’s *t* test, P < 0.0001). The weight of male pupae (Control (mean ± s.d.): 47.73 ± 7.95 mg vs. Treatment: 62.86 ± 5.42 mg; Student’s *t* test, P < 0.0001) and weight of female pupae (Control (mean ± s.d.): 87.00 ± 8.92 mg vs. Treatment: 107.17 ± 13.29 mg; Student’s *t* test, P < 0.0001) were also significantly higher in the Treatment than in the Control (Fig. 1). *E*. *obliqua* survival rates were also significantly higher in the Treatment than in the Control (Student’s *t* test, P < 0.0001; Fig. 2). It is noteworthy that there were no significantly differences in the fitness of *E*. *obliqua* between the Antibiotic and Treatment (except index of weight of female pupae). The weight of female pupae (Antibiotic (mean ± s.d.): 97.00 ± 6.58 mg vs. Treatment: 107.17 ± 13.29 mg; Student’s *t* test, P < 0.0001) were significantly higher in the Treatment than Antibiotic. Quantitative real-time PCR analysis showed that 16 S rRNA gene expression in the Control was significantly higher (750.02 ± 120-fold) than that of the Antibiotic (Student’s *t* test, *P* < 0.0001), indicating that the gut of larvae treated with antibiotics is relatively aseptic.

### *E*. *obliqua* larval gut bacterial community composition and structure in response to EGCG

**Figure 3:**
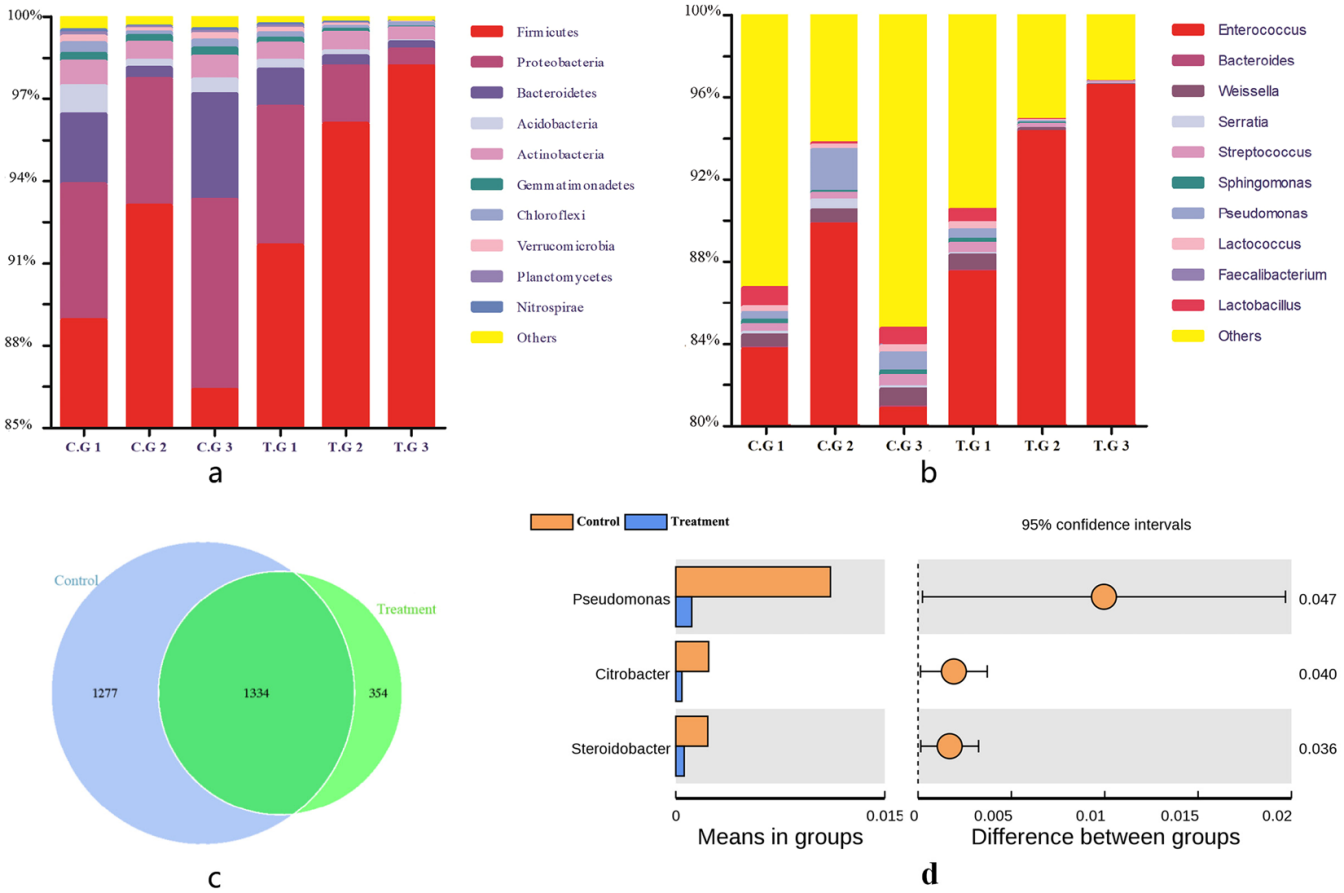
Gut bacterial community structure variation of *E*. *obliqua* larvae (n = 3) (**a**) at phylum level; (**b**) genus level; (**c**): operational taxonomic unit (OTU) level; and (**d**) Relative abundances of the top 30 genera that showed significant differences among samples from Control and Treatment. Students *t* test was used to evaluate the significance of differences between two groups. *P < 0.05; **P < 0.001; ***P < 0.0001. C.G. = Control group: larvae fed on artificial diet; T.G. = Treatment group: larvae fed on artificial diet with 1% EGCG supplement.

*Firmicutes* was the dominant phylum in both groups [Control (mean ± s.d.): 89.48 ± 2.80% vs. Treatment: 95.32 ± 2.73%], followed by *Proteobacteria* [Control (mean ± s.d.): 5.50 ± 1.03% vs. Treatment: 2.60 ± 1.85%], and *Bacteroidetes* [Control (mean ± s.d.): 2.27 ± 1.41% vs. Treatment: 0.66 ± 0.50%] (Fig. 3a). At the genus level, *Enterococcus* was the dominant genus in the Control (84.80 ± 3.73%) followed by *Pseudomonas* (1.10 ± 0.70%), *Weissella* (0.72 ± 0.11%), and *Streptococcus* (0.42 ± 0.09%). For the treatment group, *Enterococcus* was the dominant genus (92.79 ± 3.85%), followed by *Weissella* (0.32 ± 0.33%), *Bacteroides* (0.25 ± 0.16%), and *Streptococcus* (0.25 ± 0.18%) (Fig. 3b). *Enterococcus* was dominant in the guts of both the Control and Treatment group and there was no significant difference in dominant bacterial species between the larvae of the two groups. There was a large degree of overlap in gut bacterial communities between the two groups, with 1334 OTUs shared by the two groups (Fig. 3c). Common OTUs accounted for approximately 79% of the OTUs in the Treatment. It is noteworthy that the *Pseudomonas* in the Control was significantly higher than that in the Treatment (Fig. 3d, Student’s *t* test, P < 0.05). Detailed data are shown in the attached table (Table S2).

### Effects of EGCG on gut bacterial diversity and abundance of *E*. *obliqua* larvae

**Figure 4:**
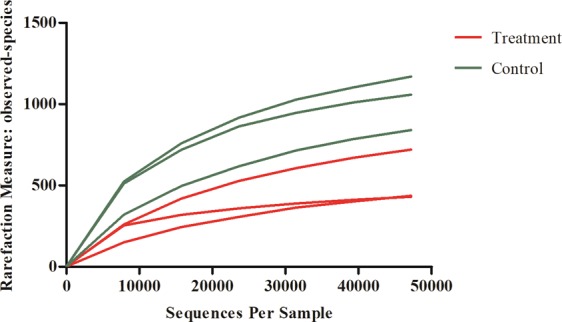
Rarefaction curves of six gut samples based on Hiseq sequencing of *E*. *obliqua* larval gut bacterial communities. Control group: larvae fed on artificial diet; Treatment group: larvae fed on artificial diet with 1% EGCG supplement.

In the six representative *E*. *obliqua* larval gut samples, we obtained a total of 432,290 sequences [99.2% of the total trimmed sequences (435,922)], which grouped into 2965 operational taxonomic units (OTUs) at 97% similarity cutoff level. Rarefaction curves of the six samples almost reached equilibrium, indicating that the natural bacteria diversity was well represented by the sequencing analysis (Fig. 4).

Alpha diversity was estimated using five measurements: number of OTUs, ACE index, Chao1 index, Shannon–Weiner index, and Simpson’s index (Table 1). Alpha diversity differed significantly between the Control and Treatment larvae gut bacterial communities in all of the calculated indices except for Simpson’s diversity which was not statistically significant.

**Table 1:** Comparison of diversity indices (mean ± SD, *n* = 3) of *E*. *obliqua* gut bacterial communities between two groups.

Index	Control group	Treatment group	*P* value^a^
Number of OTUs	1025.00 ± 115.28	450 ± 19.71	0.0038**
ACE diversity	1268.11 ± 126.70	577.61 ± 75.37	0.0027**
Chao 1 diversity	1189.26 ± 125.37	563.93 ± 53.87	0.0029**
Shannon diversity (H)	2.91 ± 0.75	1.05 ± 0.23	0.0289*
Simpson’s diversity	0.21 ± 0.02	0.40 ± 0.10	0.07

OTU richness and diversity values were calculated at genus-level. ^a^An unpaired two-tailed *t*-test was performed to assess the significance of differences between groups unless otherwise stated. *P < 0.05; **P < 0.001; ***P < 0.0001. Control group: larvae fed on artificial diet; Treatment group: larvae fed on artificial diet with 1% EGCG supplement.

**Figure 5:**
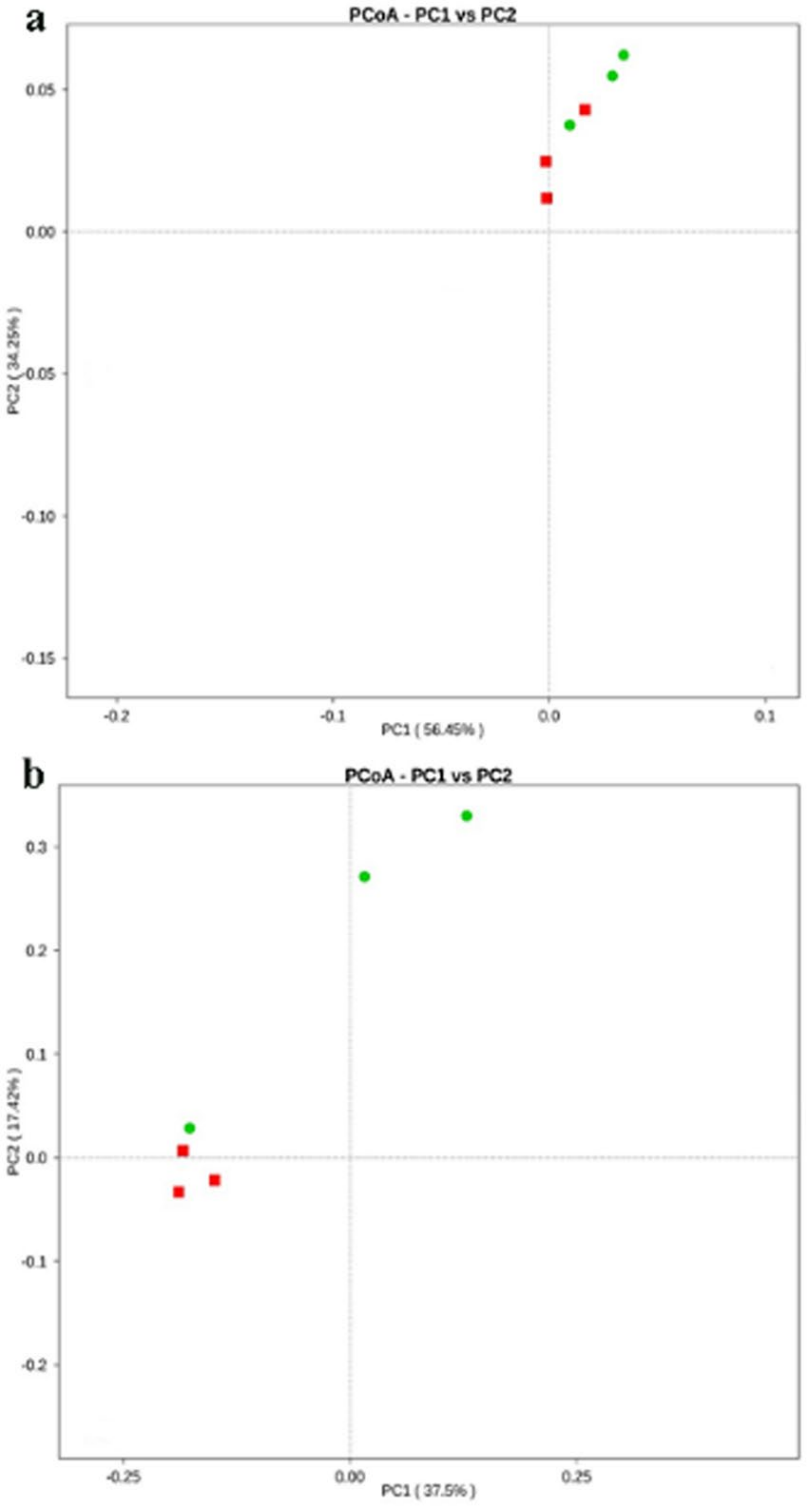
Principal coordinates analysis of differences in gut bacteria of *E*. *obliqua* larvae between control and EGCG treatment groups (PERMANOVA, *P* < 0.05). (**a)** the Principal coordinate analysis (PCoA) using weighted UniFrac distance; (**b)** the Principal coordinate analysis (PCoA) using unweighted UniFrac distance. Red symbols represent samples from control group’s larvae, and green symbols represent samples from EGCG treatment group’s larvae.

The PCoA using weighted unifrac distance showed no significant differences in β- diversity of the bacterial communities among larval gut bacteria of the two groups (Fig. 5a). In contrast, the PCoA using unweighted unifrac showed statistically significant differences among the two groups (Fig. 5b).

**Figure 6:**
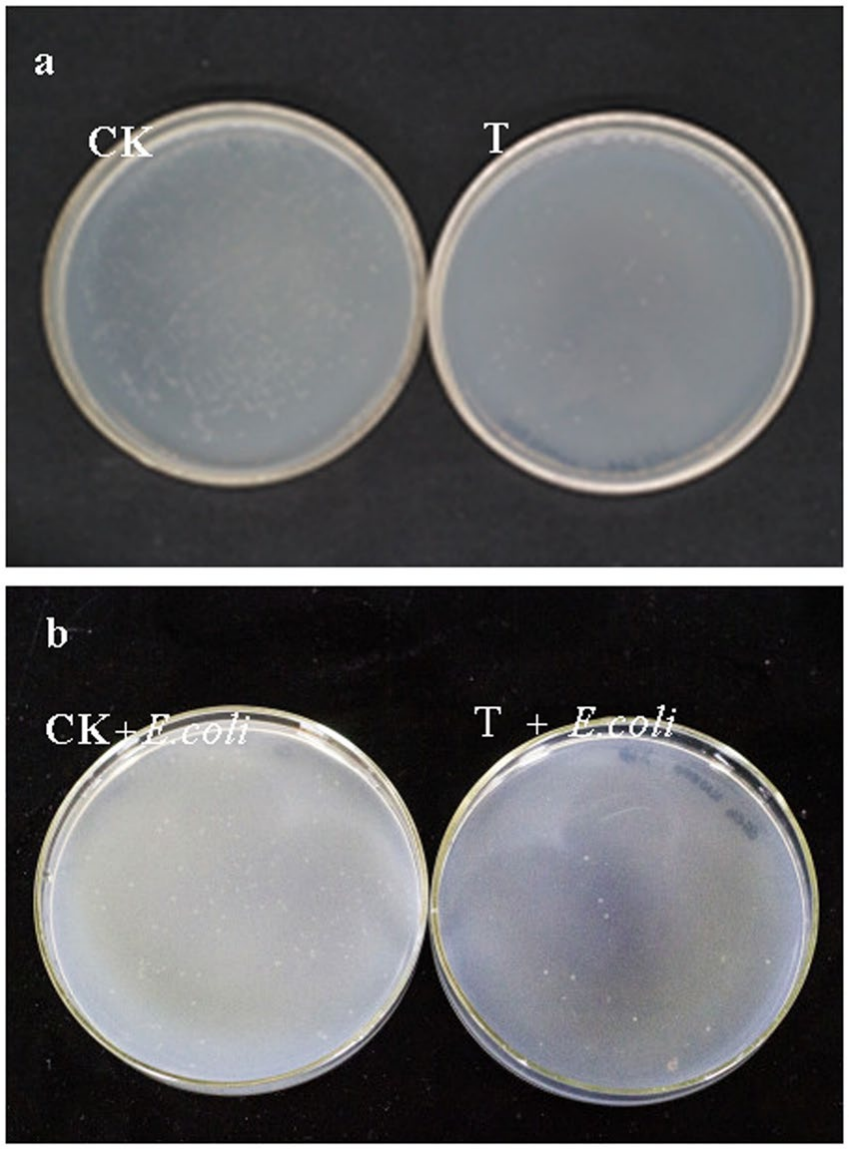
CFU analysis of difference in cultivable gut bacteria of *E*.*obliqua* larvae between control groups and EGCG treatment groups (n = 5). **(a**) Dilute to 1 × 10^5^ times. CK: larvae feed on artificial diet; T: larvae feed on artificial diets containing 1% EGCG; (**b**) Dilute to 1 × 10^4^ times. CK + *E*. *coli*: After larvae feed on artificial diets five days, we switched to feed on artificial diets containing 20 ml *Escherichia coli* for 24 hours; T + *E*. *coli*: after larvae feed on artificial diets containing 1% EGCG five days, we switched to feed on artificial diets containing 20 ml *Escherichia coli* for 24 hours.

Quantitative real-time PCR analysis showed that 16 S rRNA gene expression in the control group was significantly higher (8.02 ± 0.93-fold) than that of the treatment group (Student’s *t* test, *P* < 0.0001), indicating that the total quantity of gut bacteria was higher in the control group’s larvae. CFU analysis showed a similar result (Fig. 6). EGCG treatment reduced the number of culturable bacteria in the gut of *E*. *obliqua* larvae from 2.92 × 10^6^ CFU/ml to 7.08 × 10^4^ CFU/ml (Table 2).

**Table 2:** CFU analysis of difference in cultivable gut bacteria of *E*.*obliqua* larvae between control groups and EGCG treatment groups (n = 5). (10^−3^, 10^−4^, 10^−5^) are dilution mutiples; CK: larvae feed on artificial diet; T: larvae feed on artificial diets containing 1% EGCG; CK + *E*. *coli*: After larvae feed on artificial diets five days, we switched to feed on artificial diets containing 20 ml *Escherichia coli* for 24 hours; T + *E*. *coli*: after larvae feed on artificial diets containing 1% EGCG five days, we switched to feed on artificial diets containing 20 ml *Escherichia coli* for 24 hours.

Lable	10^−3^	10^−4^	10^−5^	Total (CFU/ml)
CK 1	countless	1666	150	2.92 × 10^6^
CK 2	countless	1450	135	
CK 3	countless	1245	145	
CK 4	countless	1877	141	
CK 5	countless	999	158	
T 1	500	43	uncounted	7.08 × 10^4^
T 2	457	33	uncounted	
T 3	380	36	uncounted	
T 4	280	32	uncounted	
T 5	340	33	uncounted	
CK + *E*. *Coli* 1	1200	77	uncounted	4.8 × 10^5^
CK + *E*. *Coli* 2	1100	99	uncounted	
CK + *E*. *Coli* 3	1300	98	uncounted	
CK + *E*. *Coli* 4	999	104	uncounted	
CK + *E*. *Coli* 5	1000	103	uncounted	
T + *E*. *Coli* 1	100	uncounted	uncounted	1.02 × 10^4^
T + *E*. *Coli* 2	99	uncounted	uncounted	
T + *E*. *Coli* 3	98	uncounted	uncounted	
T + *E*. *Coli* 4	105	uncounted	uncounted	
T + *E*. *Coli* 5	111	uncounted	uncounted	

## Discussion

All fitness measures were improved in the Treatment as compared with the Control, indicating that EGCG may have a significant impact on fitness of *E*. *obliqua* larvae. And there was no significantly difference of all fitness measures (except index of female pupae weight) between Treatment and Antibiotic, indicating that EGCG promotes the fitness of larvae may be related to the antibacterial function of EGCG. Through observation we found that the Control was more susceptible to bacterial infection and had higher mortality. Previous study shown that EGCG plays an important role in antibacterial function^19^. Therefore, we believe that EGCG improves the fitness of *E*. *obliqua* larvae due to its antibacterial function.

Using next generation sequencing technology, we assessed changes in the diversity and richness of gut bacterial communities of *E*. *obliqua* larvae after EGCG treatment. The EGCG treatment group had significantly lower bacterial species richness and evenness than the control group for three of the four alpha diversity indices. However, the dominant bacteria of the two groups were similar, and included *Enterococcus*, *Weissella*, *Bacteroides*, *Lactococcus*, *Serratia*, and *Pseudomonas*. These bacteria help to improve nutrient compositions of nutrient-poor host insect diets, aid digestion of difficult to digest food components, protect the host from predators, parasites, and pathogens, and are involved in host mating and reproductive^20^. For example, *Enterococcus* is active throughout the larval life cycle in most Lepidoptera larval guts, and probably plays a key role in insect defense against potentially harmful microorganisms^21^. Meanwhile, the phenomenon that gut bacteria promote the fitness of host insects is not unchangeable. As an example, *Pseudomonas* of larvae gut of the coffee berry borer beetle *Hypothenemus hampei* (Coleoptera: Curculionidae), which can help host degradation of the caffeine^22^. And for other insects, the *Pseudomonas* is generally considered to be an insect pathogen^23^. Therefore, inhibition of colonization of *Pseudomonas* by EGCG in gut may be one of the reasons for promoting larval fitness.

Caterpillars lack a resident gut microbiome and the number of bacteria in the gut of Lepidoptera larvae is much lower than that of other insects or animals^24^. And many of bacterial in the gut of caterpillars are transient bacterial, short-lived in the gut of healthy animals, are metabolized in the intestine to produce substances that affect the healthy growth of the host^25^. In our study we demonstrated that EGCG could inhibits colonization by many bacterial species (e.g., transient microbes),such as *Pseudomonas*, *Citrobacter* and *Terrimonas*, of larval guts, and promoting larval fitness. Using q-RT-PCR technology and the CFU method, we also assessed changes in the abundance of gut bacteria in *E*. *obliqua* larvae guts after EGCG treatment. We found that EGCG treatment can significantly reduce the number of gut bacteria in larvae. The number of gut bacteria in larvae following EGCG treatment was close to that reported previously (Geometridae: 1 × 10^3^–1 × 10^5^). In short, we think that one of the reasons why EGCG promotes larval fitness is that it inhibits the colonization of a large number of bacteria in the gut which is confirmed by the results of antibiotic treatment.

Because of longstanding co-evolution, *E*. *obliqua* larvae have adapted to secondary compounds found in tea leaves, and may even benefit from these compounds, as is the case for EGCG. Therefore, this study sheds light on one of reasons underlying plant–insect interactions and benefits derived by herbivorous insects, even pests, from their host plants.

## Materials and Methods

### Insect collection and maintenance

400 *E*. *obliqua* eggs were obtained from stock cultures from the State Key Laboratory of Tea Plant Biology and Utilization, Anhui Agricultural University, Hefei, China (31.86′N, 117.27′E). *E*. *obliqua* were reared on tea leaves at temperatures of 22 ± 1°C, relative humidity of 75 ± 10%, and 16 h light:8 h dark photoperiod.

### Diet preparation

Artificial diet, 78 ml water, 12 g wheat germ, 4.5 g soy flour, 2 g sugar, 2 g wheat agar, 0.5 g wesson’s salt, 0.25 g methyl 4-hydroxybenzoate, 0.15 g sorbic acid, 0.1 g cholesterol, 0.05 g, and 0.04 g inositol sterilized by autoclave, 0.2 g Vitamin C, 0.2 g yeast, 0.05 g and choline chloride solution sterilized by membrane filtration and added after artificial diet temperature was lower than 60°C. EGCG treated agar-based artificial diet, where 1% EGCG (989-51-5; EBIKAR, Hangzhou, China) solution was sterilized by membrane filtration and added after artificial diet temperature was lower than 30°C, and antibiotic treated agar-based artificial diet, where antibiotic solution (300 µg/mL each rifampicin, gentamicin, penicillin, and streptomycin) was sterilized by membrane filtration and added after artificial diet temperature was lower than 60°C.

### Insect rearing and EGCG treatment

Larvae were reared on tea leaves until their third larval instar (III−1). 210 healthy third instar larvae were equally divided into three groups and placed individually in 90 mm diameter plastic Petri dishes with tight lids containing insect diet. The control group (Control) was fed artificial diet, the treatment group (Treatment) was fed the EGCG treated agar-based artificial diet, and the antibiotic treatment group (Antibiotic) was feed the antibiotic treated agar-based artificial diet. There were 7 replicates in each group and 10 larvae in each replicate. The three groups of *E*. *obliqua* larvae were then reared until the adult stage under these conditions.

To analyze the effect of EGCG treatment on the fitness of *E*. *obliqua*, we recorded the following fitness indicators of the three groups of larvae: survival rate, pupa weight (male and female), and eclosion rate.

### DNA preparation, PCR, high-throughput sequencing, and quantitative real-time PCR of *E*. *obliqua* gut bacteria complex

For DNA extraction of the gut bacteria complex of *E*. *obliqua* larvae, fifth instar larvae (V-1, n = 3) were surface sterilized by dipping them in 70% ethanol once (about 15 s) and then in sterile water rinse twice (about 30 s). Dissecting scissors were used to cut laterally behind the head capsule, and the gut was removed from the cuticle with larval forceps. The whole gut including gut contents was collected and placed in a 2.0 ml micro-centrifuge tube for processing (all steps were performed on ice). Samples were placed in a −80 °C freezer. Total genome DNA was extracted from samples using QIAamp DNA Stool Mini Kit (Qiagen, USA). DNA concentration and purity were monitored on 1% agarose gels. DNA was diluted to 1 ng/μL using sterile water.

For amplicon generation, a 450-bp fragment of 16 S rRNA from the 16 S V4 region was amplified using the specific primer pair 515F-806R (Table S1). All PCR reactions were carried out with Phusion® High-Fidelity PCR Master Mix (New England Biolabs). We mixed the same volume of 1 × loading buffer (containing SYB green) with PCR amplicons and conducted electrophoresis on 2% agarose gel for detection. Samples with a bright band of between 400–450 bp were chosen for further experiments. PCR amplicons were mixed in equal density ratios. Then, the PCR amplicon mixture was purified with Qiagen Gel Extraction Kit (Qiagen, Germany).

For library preparation and sequencing, sequencing libraries were generated using TruSeq® DNA PCR-Free Sample Preparation Kit (Illumina, USA) following manufacturer’s recommendations and index codes were added. The library quality was assessed on the Qubit@ 2.0 Fluorometer (Thermo Scientific) and Agilent Bioanalyzer 2100 system. The library was sequenced on an Illumina HiSeq. 2500 platform and 250 bp paired-end reads were generated.

Total RNA of samples from the two groups was extracted using an SV total RNA isolation system with a DNase purification step (Promega) following the manufacturer’s instructions. A 2 μg RNA sample was reverse transcribed using the PrimeScript™ RT Master Mix (Takara, Shiga, Japan).

Quantitative real-time PCR (q-RT-PCR) was performed using an ABI 7300 Real-Time PCR System (Applied Biosystems, Foster City, CA, USA) using GoTaq qPCR Master Mix (Promega) at a volume of 10 μL. The q-RT-PCR was performed under the following conditions: 95 °C for 30 s, followed by 40 cycles of 95 °C for 5 s and 60 °C for 30 s, and q-RT-PCR data were collected and analyzed via the 2^−ΔΔCt^ method^26^. All samples were independently measured three times. The forward and reverse primers used for the genes of interest are described in supplemental Table S1. The *β*-actin gene was used as a reference for gene expression normalization^27^.

### Bioinformatics and statistical analysis

After paired-end reads assembly and quality control, sequence analysis was performed using Uparse software (Uparse v7.0.1001)^28^. Sequences with ≥97% similarity were assigned to the same operational taxonomic units (OTUs). A representative sequence for each OTU was screened for further annotation. For each representative sequence, the RDP classifier (Version 2.2) algorithm was used to search the GreenGene Database and to annotate taxonomic information^29,30^. To study phylogenetic relationships among the OTUs and compare dominant species in different samples (groups), multiple sequence alignment was conducted using MUSCLE software (Version 3.8.31).

Abundance values of OTUs were normalized using a standard of sequence number corresponding to the sample with the least sequences. Subsequent analysis of alpha diversity and beta diversity were performed using this normalized output. Alpha diversity of the gut bacteria complexes was calculated as observed species richness, two richness estimators [the abundance-based coverage estimator (ACE) and a nonparametric richness estimator based on distribution of singletons and doubletons (Chao 1)], and two diversity indices (Shannon–Wiener and Simpson’s index). Rarefaction curves were estimated using the ‘alpha_rarefaction.py’ script in QIIME at 97% similarity and a 47,194 cutoff. Rarefaction curves were generated based on observed species richness. Diversity indices of the treatment and control groups were compared using Tukey test and paired Wilcoxon’s test.

Beta diversity analysis was used to evaluate differences in species complexity among the two groups. Beta diversity of both weighted and unweighted unifrac were calculated using QIIME software (Version 1.7.0). Principal Coordinate Analysis (PCoA) was performed to visualize the gut bacteria species complex, and to illustrate the differences in the larval gut bacterial community composition and structure on the unifrac distances of the unweighted and weighted distance matrices, respectively.

### Colony-Forming Units (CFU) and infection of *E*. *coli*

To determine which of the gut bacterial community could form colonies, we first extracted whole larval guts under sterile condition, as described previously. Next, the whole gut was homogenized in sterilized PBS and diluted with sterile water into five concentration gradients (10^−1^, 10^−2^, 10^−3^, 10^−4^, and 10^−5^). Then, gut homogenates were poured on bacteria culture medium (3 g beef extract, 10 g peptone, 5 g NaCl, 20 g agar and 1000 ml water, pH 7.4). The bacteria culture media were incubated at 37 °C for 48 h. Finally, the CFU was calculated (n = 5)^31^.

In order to verify the antibacterial function of EGCG. *Escherichia coli* was obtained from School of Life Sciences, Anhui Agricultural University, Hefei, China and maintained at 4 °C. The strain was grown in Luria Broth (LB) medium, adjusted to an optical density (OD_600_) of 0.74. After larvae (n = 5) were fed for five days, we switched to feed on artificial diets containing 20 ml *E*. *coli* for 24 hours. We then calculated the number of gut bacteria using the CFU method.

## Supplementary information

Positive effects of the tea catechin (-)-epigallocatechin-3-gallate on gut bacteria and fitness of Ectropis obliqua Prout (Lepidoptera: Geometridae)

Table S2
