# Supplementary material for: Positive effects of the tea catechin (-)-epigallocatechin-3-gallate on gut bacteria and fitness of Ectropis obliqua Prout (Lepidoptera: Geometridae)
Source: Sci Rep. 2019 Mar 22;9:5021. doi: 10.1038/s41598-019-41637-9 (PMC6430822; doi:10.1038/s41598-019-41637-9)
Supplement: Supplementary file 1 — Positive effects of the tea catechin (-)-epigallocatechin-3-gallate on gut bacteria and fitness of Ectropis obliqua Prout (Lepidoptera: Geometridae) [file 41598_2019_41637_MOESM1_ESM.docx]

**Positive effects of the tea catechin (-)-epigallocatechin-3-gallate on gut bacteria and fitness of *Ectropis obliqua* Prout (Lepidoptera: Geometridae)**

Yong Zhang^1^. Tianyu Zhao^1^. Jundan Deng^2^. Xiaomin Zhou^1^. Zhenzhen Wu^3^. Qingqing Su^3^ . Longwa Zhang^2^_._ Yanhua Long^3*^_._ Yunqiu Yang^1*^.

^1^State Key Laboratory of Tea Plant Biology and Utilization, Anhui Agricultural University, Hefei 230036;

^2^School of Forestry and Landscape Architecture, Anhui Agricultural University, Hefei 230036, China

^3^School of Life Sciences, Anhui Agricultural University, Hefei 230036

***Correspondence: E-mail: yyq_lyh@ahau.edu.cn; longyanhua@ahau.edu.cn

Tel: 86-10-65786401

**Table S1** Primer sequences used in this study

| Primer name | Primer sequence (5’-3’) |
| --- | --- |
| 16SF | TCCTACGGGAGGCAGCAGT |
| 16SR | GGACTACCAGGGTATCTAATCCTGTT |
| 16S-515F | GTGYCAGCMGCCGCGCTAA |
| 16S-806R | GGACTACHVGGGTWTCTAAT |
| *β*-actinF | GCTTCTCCTTGATGTCACGCAC |
| *β*-actinR | CCGGTCGTACCACCGGTATC |

**Table S2** Relative abundances of the genera level that showed significant differences among samples from Control and Treatment. Students *t* test was used to evaluate the significance of differences between two groups. group1: Control; group2: Treatment. *P<0.05; **P<0.001;***P<0.0001.
